# Polyacrylate-GnRH Peptide Conjugate as an Oral Contraceptive Vaccine Candidate

**DOI:** 10.3390/pharmaceutics13071081

**Published:** 2021-07-15

**Authors:** Mohammad O. Faruck, Prashamsa Koirala, Jieru Yang, Michael J. D’Occhio, Mariusz Skwarczynski, Istvan Toth

**Affiliations:** 1 School of Chemistry and Molecular Biosciences, The University of Queensland, St. Lucia, Brisbane, QLD 4072, Australia; m.faruck@uq.edu.au (M.O.F.); p.koirala@uq.edu.au (P.K.); jieru.yang@uq.edu.au (J.Y.); 2 School of Life and Environmental Sciences, The University of Sydney, Sydney, NSW 2006, Australia; michael.docchio@sydney.edu.au; 3 School of Pharmacy, The University of Queensland, Woolloongabba, Brisbane, QLD 4102, Australia; 4 Institute for Molecular Bioscience, The University of Queensland, St. Lucia, Brisbane, QLD 4072, Australia

**Keywords:** immunocontraceptive, GnRH, oral vaccine, poly(methylacrylate), pig T helper, PADRE, antibody

## Abstract

Contraceptive vaccines are designed to elicit immune responses against major components of animal reproductive systems. These vaccines, which are most commonly administered via injection, typically target gonadotropin-releasing hormone (GnRH). However, the need to restrain animals for treatment limits the field applications of injectable vaccines. Oral administration would broaden vaccine applicability. We explored contraceptive vaccine candidates composed of GnRH peptide hormone, universal T helper PADRE (P), and a poly(methylacrylate) (PMA)-based delivery system. When self-assembled into nanoparticles, PMA-P-GnRH induced the production of high IgG titers after subcutaneous and oral administration in mice. PADRE was then replaced with pig T helper derived from the swine flu virus, and the vaccine was tested in pigs. High levels of systemic antibodies were produced in pigs after both injection and oral administration of the vaccine. In conclusion, we developed a simple peptide–polymer conjugate that shows promise as an effective, adjuvant-free, oral GnRH-based contraceptive vaccine.

## 1. Introduction

Invasive animal pest species cause major negative environmental impacts. For example, wild boars, which are the world’s major invasive pest species, pose a serious threat to biosecurity globally. They are a large reservoir of diseases of domestic pigs, which creates a significant risk to commercial pig production. Wild boars also reduce agricultural productivity and cause major ecological damage. The control of wild boar is made difficult by their large reproductive capacity and wide dispersal across both occupied and unoccupied landscapes. Invasive animals can be controlled by lethal or non-lethal methods [1]. Lethal methods typically include culling of animals [1,2]. Non-lethal approaches include fertility control or relocation. Fertility controls decrease the number of invasive animals by reducing their ability to reproduce [3,4]. Reproductive control methods are mainly divided into surgical and non-surgical. Castration is the most common form of surgical sterilization but can be associated with morbidity and mortality, and is often impractical [5,6]. Non-surgical methods are therefore preferable for population control in invasive pest, as well as domesticated, animals. Of these, immunocontraceptive vaccines are one of the most promising strategies [7,8]. Immunocontraception is designed to neutralize the biological action of reproductive hormones, typically gonadotropin releasing hormone (GnRH) [9]. Vaccines against GnRH have been developed to improve meat quality in production animals (e.g., pork) and to reduce aggression in domestic animals [10,11].

Gonadotropin-releasing hormone (GnRH), also known as luteinizing hormone-releasing hormone (LHRH), is secreted by the hypothalamus [12,13,14]. Its main role is to regulate follicle-stimulating hormone (FSH) and luteinizing hormone (LH) secretion from the pituitary gland [14,15]. LH stimulates gonadal steroid secretion, which impacts fertility and reproduction in both male and female animals. In males, LH stimulates testosterone secretion from Leydig cells, while in females, it promotes ovulation and progesterone secretion [14,15].

The injectable GnRH vaccine GonaCon™ has been used to suppress fertility in feral, wild and domestic animals, including white-tailed deer [16], female cats [11], and female wild boar [17,18,19]. GonaCon™ relies on the adjuvant AdjuVac™ to induce an immune response [20,21]. AdjuVac is derived from killed *Mycobacterium avium*, so vaccinated animals are often falsely test positive to tuberculosis, which is a major disadvantage for farm animals [22]. Adverse effects of GonaCon™ have been reported, including granulomatous nodules and sterile abscesses at the injection site and lymph nodes [16,23]. In addition, the practicality of GonaCon™ and other injectable contraceptive vaccines is limited for wild and invasive pest animals, which must be captured and restrained for injection. There is an urgent need therefore for a vaccine that can be delivered orally, and without the need for bacteria-derived adjuvants.

**Figure 1:**
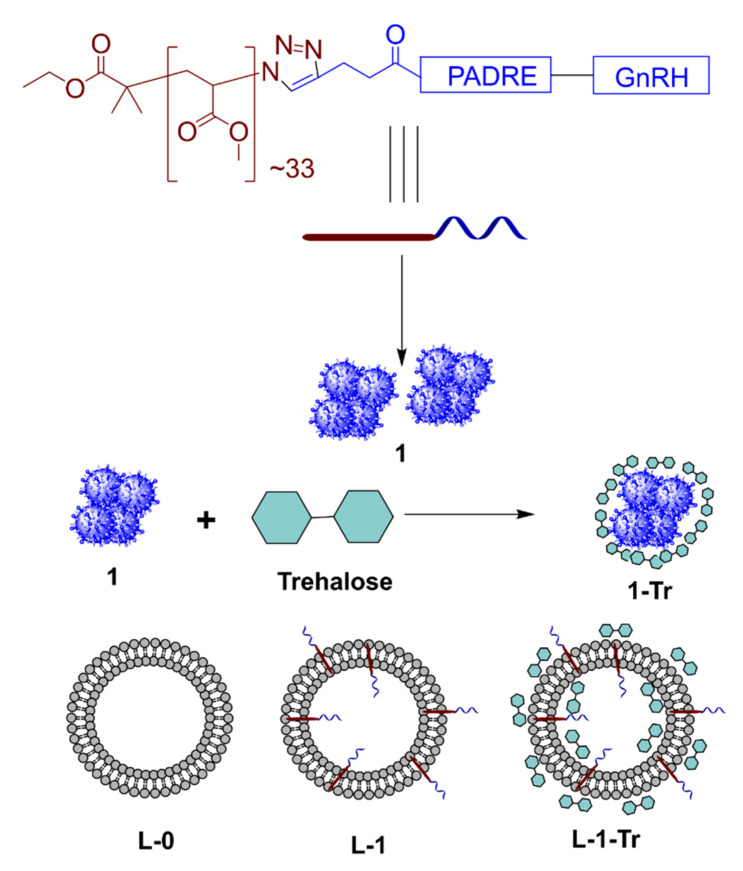
Vaccine candidate **1** and its formulations: **1-Tr**—Conjugate **1** formulated with trehalose; **L-O**—Blank liposomes; **L-1**—Conjugate **1** anchored to liposomes; **L-1-Tr**—Conjugate **1** anchored to liposomes and formulated with trehalose.

Both linear and branched polyacrylate polymers have been used widely in vaccine delivery systems to induce systemic humoral [24,25,26] and cellular [27,28,29] immune responses. Furthermore, it was recently demonstrated that polyacrylate-based Group A Streptococcus vaccine can be delivered orally [30]. With the aim of developing an orally administrable contraceptive vaccine, we designed a conjugate system composed of GnRH (EHWSYGLRPG), universal T helper PADRE (AKFVAAWTLKAAA) and poly(methylacrylate) (Figure 1). We also designed polyacrylate-based vaccine bearing pig T helper (KHKVRDEVMVHWFDD) derived from swine fever virus. The polymer peptide-conjugates were examined in vivo alone, and in liposomal formulations, following subcutaneous and oral administration.

## 2. Materials and Methods

### 2.1. Materials

All chemicals used were analytical grade. Protected Fmoc amino acids and rink amide p-methylbenzhydrylamine (MBHA) resin were purchased from Novabiochem (Laufelfingen, Switzerland). 1-[Bis(dimethylamino)methylene]-1H-1,2,3-triazolo[4,5-b] pyridinium3-oxid hexafluorophosphate (HATU) was purchased from Minotopes (Melbourne, Australia). *N*,*N*-diisopropylethylamine (DIPEA), *N*,*N*-dimethylformamide (DMF), dicholoromethane (DCM), piperidine, trifluroacetic acid (TFA), and acetonitrile were purchased from Merck (Hohbrunn, Germany). 1,2-Dihexadecanoyl-sn-glycero-3-phosphocholine (DPPC), dimethyldioctadecylammonium bromide (DDAB), cholesterol, an Avanti mini extruder, PC membranes and filter support were obtained from Avanti (Alabaster, AL, USA). Pentanoic acid, poly(methyl acrylate)-azide terminal (PMA), triisopropylsilane (TIS), phosphate buffer saline (PBS), horseradish peroxidase-conjugated goat anti-mouse secondary antibody IgG, and O-phenylenediamine dihydrochloride (OPD) substrate were purchased from Sigma Aldrich (St. Louis, MO, USA).

### 2.2. Equipment

Peptide synthesis was performed by microwave-assisted Fmoc (9-fluorenylmethyloxycarbonyl) solid-phase peptide synthesis (SPPS) using a CEM Discover microwave with Synergy software, Version DE 10.3. Electrospray ionization-mass spectrometry (ESI-MS) was conducted on a Perkin-Elmer-Sciex API3000 instrument with Analytes 1.4 software (Applied Biosystem/MDS Sciex, Toronto, ON, Canada). Analytical HPLC was performed on DGU-20A3, LC-20AB, SIL-20ACHT, and SPmD-M10AVP Shimadzu instruments (Kyoto, Japan), with a flow rate of 1 mL/min and detection at 214 nm. Analytical HPLC analysis was done using 0–100% solvent B for 50 min (0.1% TFA in water) and solvent A (90% MeCN, 10% water and 0.1% TFA) on a Vydac analytical C18 column (218TP54; 5 µm, 4.6 mm × 250 mm). Preparative HPLC was performed on CBM-20A, SPD-20A, LC-20AP and FRC-10A Shimadzu (Tokyo, Japan) with a Vydac C18 column at a 20 mL/min flow rate and detection at 230 nm. Particle size was measured by dynamic light scattering (DLS) using a Zetasizer, (Nano Science Series ZS, Malvern Instruments, Version number: MAN0383-06-EN, Malvern, UK) with DTS software. Transmission electron microscopy (TEM; HT7700 Exalens, Hitachi Ltd., Tokoyo, Japan) was performed at the Australian Microscopy & Microanalysis Research Facility within the Centre for Microscopy and Microanalysis, The University of Queensland (UQ). Element microanalysis was performed at the School of Chemistry and Molecular Biosciences, UQ (FLASH 2000, Thermo Fisher Scientific, Waltham, MA, USA).

### 2.3. Synthesis of Peptides, PADRE-GnRH with Alkyne Moiety (P-GnRH)

P-GnRH (pentynoyl-AKFVAAWTLKAAA-EHWSYGLRPG) was synthesized using Fmoc-SPPS on a 0.2 m·mol scale in a CEM Discover microwave with Synergy software, following a previously established protocol [31,32]. Resin was pre-swelled in DMF overnight. Before synthesis began, Fmoc was deprotected twice (5 min, then 10 min) with 80% DMF and 20% piperidine. All amino acids in 4.2 equiv. were activated by 0.5M HATU in DMF (4.0 equiv. 800 µL) and DIPEA (5.2 equiv. 91 µL) as a neutralizing base. Each amino acid was coupled twice, for 10 min, then 15 min. The process was repeated until the desired sequence was obtained. 4-pentynoic acid was then coupled twice (15 min, then 20 min) to the N-terminus of the peptide. The resin was washed with DMF, DCM, and methanol and kept in a desiccator for drying overnight. The peptide was cleaved from the resin using a mixture of trifluroacetic acid (TFA, 95%), triisopropylsilane (TIPS, 2.5%), and water (2.5%). TFA was removed under reduced pressure and the peptide was precipitated in cold diethyl ether, then dissolved in acetonitrile:water (50:50) solution and purified using preparative HPLC (C18 column). Molecular weight = 2612.99. ESI-MS [M + 2H]^2+^ *m*/*z* 1306.1 (calc. 1307.0), [M + 3H]^3+^ *m*/*z* 871.0 (calc. 871.6), [M + 4H]^4+^ *m*/*z* 654.6 (calc. 654.0). HPLC, C18 column, *t*_R_ 20.1 min, purity 96%, yield 43% (Supporting Information Figure S1).

### 2.4. Synthesis of Pig T helper-GnRH with Alkyne Moiety (PT-GnRH)

PT-GnRH (pentynoyl-KHKVRDEVMVHWFDD-EHWSYGLRPG) was synthesized analogously to PADRE-GnRH except pentynoic acid was coupled to the peptide N-terminus instead of the acetyl group. The crude compound was purified using preparative HPLC (C18 column). Molecular weight = 3203.63, ESI-MS [M + 2H]^2+^ *m*/*z* 1602.3 (calc. 1602.8), [M + 3H]^3+^ *m*/*z* 1068.5 (calc. 1068.8), [M + 4H]^4+^ *m*/*z* 801.5 (calc. 801.9). HPLC C18 column, *t*_R_ 19.8 min, purity 99%, yield 43% (Supporting Information Figure S2).

### 2.5. Synthesis of Polymer-Peptide Conjugates, ***1*** and ***2***

**Figure 2:**
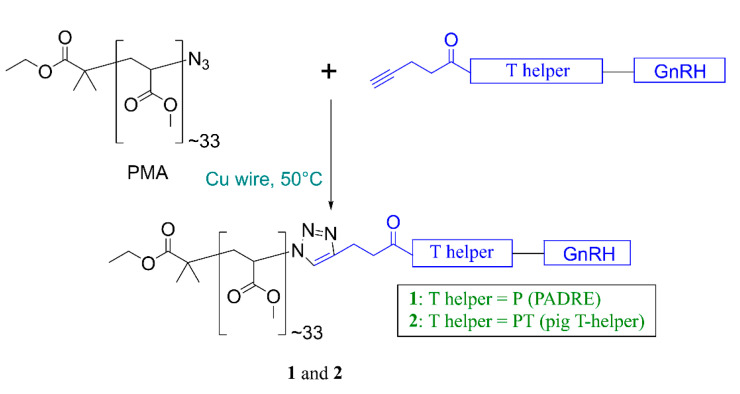
Synthesis of vaccine candidates **1** and **2**. PMA, poly(methylacrylate) azide.

P-GnRH was conjugated to poly(methyl acrylate)-azide (PMA) using copper(I)-catalyzed alkyne-azide cycloaddition (CuAAC) click reaction (Figure 2), as previously reported [25,33]. Copper wire was submerged in concentrated sulfuric acid for 1 min, then washed on a glass filter funnel with Milli Q water (5 times) and methanol (5 times) before being dried under a stream of nitrogen. PMA (1 equiv.) and P-GnRH (1.4 equiv.), copper wire, and DMF were added to the flask and stirred. The click reaction was stopped after 12 h when the solution color turned to greenish blue. The resulting solution was filtered through a Cameo^®^ syringe filter with PTFE membrane (pore size 0.45 μm, volume 12 mL). Conjugate **1** was self-assembled by solvent exchange using a syringe pump (DMF solution from **1** was slowly added to the water over three hours) [34]. Conjugate **1** was dialyzed for three days to remove unreacted peptide, copper, and organic solvent. The resulting conjugate nanoparticles were analyzed by element microanalysis to determine the substitution ratio as reported previously [24] and the nanoparticles were characterized by DLS and TEM. Compound **2** was synthesized in an identical manner to **1**.

### 2.6. Preparation of ***1-Tr***

Compound **1** was formulated with trehalose cryoprotectant. Trehalose (5 mg) was dissolved in water (1 mL) and stirred overnight. Peptide–polymer conjugate **1** (1 mg in 1 mL water) was then mixed gently with the trehalose solution. The solution was vortexed for 1 min, slowly frozen with stirring, then freeze-dried. Dried **1-Tr** was dissolved in water or PBS for further study.

### 2.7. Preparation of Liposomes, ***L-0*** and ***L-1***

Thin lipid hydration was used to produce the liposomes [35,36]. DPPC (4 mg), DDAB (0.017 mg), and cholesterol (1.05 mg) (at a molar ratio of 2:0.01:1) were dissolved in 1 mL of chloroform. **1-Tr** (1 mg) was dissolved in 1 mL of methanol and added to the lipid solution. The solvent was then slowly removed under reduced pressure using a rotatory evaporator to produce a film on the inner surface of the round bottom flask. Residual chloroform was removed under vacuum overnight. The lipids were rehydrated with water (1 mL) and swirled to produce liposomes. The liposomes were extruded 21 times in both directions using a 200 nm pc membrane filter to produce uniformly sized **L-1**. Formulation in PBS was achieved by adding 0.1 mL of 10× PBS into 0.9 mL of the liposomal formulation. Blank liposomes, **L-0**, were produced in exactly the same manner.

### 2.8. Preparation of ***L-1-Tr***

DPPC (4 mg), DDAB (0.017 mg), and cholesterol (1.05 mg) at a molar ratio of 2:0.01:1 were dissolved in 1 mL of chloroform. **1-Tr** (1 mg) was dissolved in 1 mL of methanol, then added to the lipid solution. The solvent (chloroform, methanol) was slowly removed under reduced pressure using a rotatory evaporator to produce a film on the inner surface of the round bottom flask. The residual chloroform and methanol were removed under vacuum overnight. The lipids were rehydrated with PBS (1 mL) and swirled to produce liposomes. The liposomes were extruded 21 times in both directions using a 200 nm pc membrane filter to produce uniformly sized **L-1-Tr**. The solution was slowly frozen, then freeze-dried overnight. Dried **L-1-Tr** was dissolved in water or PBS for further study.

### 2.9. Characterization of the Size, Zeta-Potential, and Morphology of the Vaccine Candidates

The intensity and polydispersity index (PDI) of the vaccine candidate nanoparticles (NPs) were examined by DLS at a back-scattering angle of 173° at 25 °C in folded capillary cuvettes. The results are presented as the average of five measurements for each vaccine candidate. TEM was used to visualize the vaccine candidates’ surface morphology. A drop of the sample (0.1 mg/mL concentration) was placed on a carbon coated grid and the particles were allowed to settle for 2 min before the excess liquid was removed with filter paper. Samples were stained with 2% uranyl acetate for 30 s, then air-dried for 5 min before imaging.

### 2.10. Subcutaneous Immunization of Mice

C57/BL6 (The University of Queensland Animal Ethics Committee (AEC), AEC Approval number: SCMB/AIBN/463/18, Approval Date: 29 November 2018) female mice (six weeks old) were housed in cages under sterile conditions. Mice were evenly divided into six groups: **1**, **1-Tr**, **L-1**, **L-1-Tr,** positive control (**1+CFA**), and negative control (PBS), with five mice in each group. Each mouse received 30 μg of **1** (in the respective formulations) in 30 μL of PBS, except for the mice in the negative control group, which received 30 μL PBS. Immunization was carried out on day 1. Blood samples were collected via tail bleed on days 0 and 14. Blood was centrifuged for 10 min at 8000 rpm. The supernatant serum was then transferred into sterile tubes and stored at −80 °C for further use.

### 2.11. Oral Immunization of Mice

Mice (as above) were evenly divided into four groups: **1**, **1-Tr**, cholera toxin B-adjuvanted positive control (**1**+CTB), and negative control (PBS), with five mice in each group. All mice, except the negative controls, received 30 μg of **1** in 30 μL water via oral gavage on day 1. Blood and serum were collected as described above.

### 2.12. Intramuscular and Oral Immunization of Pigs

Female Large White pigs (The University of Queensland Animal Ethics Committee (AEC): AEC Approval number: SCMB/520/18, Approval Date: 9 January 2019) were randomly assigned to two groups. Group 1 (*n* = 3, 92.6 ± 1.9 kg) received compound **2** (3 mg) by intramuscular injection, while Group 2 (*n* = 3, 94.3 ± 1.9 kg) received compound **2** (30 mg) by oral gavage (Supporting Information Figure S6). Pig age (live weight) was chosen to match the onset of sexual maturity. Both groups received two doses of vaccine four weeks apart. Blood samples were taken immediately before both vaccinations and final blood sample was taken two weeks after the second vaccination.

### 2.13. Determination of Antibody Titers (IgG) in Mice

GnRH-specific IgG levels were examined by enzyme-linked immunosorbent assay (ELISA). 96-well microtiter plates were coated with carbonate coating buffer (CCB) comprising 50 µg of GnRH as antigen. The plates were then blocked with 5% skim milk to reduce nonspecific binding. Serum samples were serially diluted in 0.5% skim milk, starting at 1:100, down the plate. Secondary antibody (33 µL of horseradish peroxide-conjugated anti-mouse IgG) in 100 mL of 0.5% skim milk was added to the plates. The plates were then incubated with 100 µL of OPD substrate for 20 min at room temperature. Absorbance was measured at 450 nm using a Spectra Max microplate reader. Statistical significance was assessed by one-way ANOVA followed by Tukey’s post hoc test.

### 2.14. Determination of IgG Antibody Titers in Pigs

To determine the level of anti-GnRH IgG in pigs, ELISA plates were coated with 50 µg of GnRH as antigen in carbonate coating buffer (CCB). The plates were then blocked with 1% bovine serum albumin (BSA) to reduce nonspecific binding. Serum samples were serially diluted in 0.5% skim milk, starting at 1:100, down the plate. Secondary antibody (33 µL anti-pig IgG-horseradish peroxidase (HRP)) in 100 mL of 0.1% bovine serum albumin was added to the plates. The plates were incubated with 100 µL of OPD substrate for 20 min at room temperature. Absorbances were measured at 450 nm using a Spectra Max microplate reader. Statistical significance was assessed by one-way ANOVA followed by Tukey’s post hoc test.

## 3. Results

### 3.1. Synthesis and Characterization

**Figure 3:**
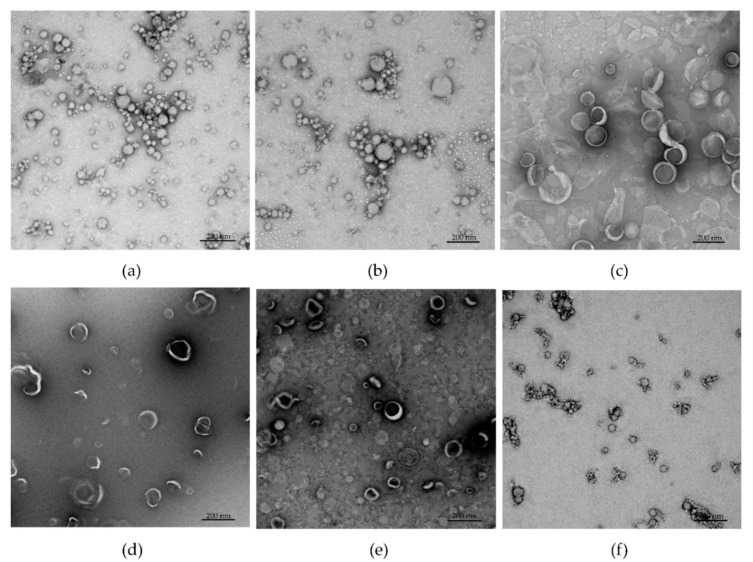
Transmission electron micrograph of (**a**) **1**, (**b**) **1-Tr**, (**c**) **L-0**, (**d**) **L-1**, (**e**) **L-1-Tr**, and (**f**) **2**, stained with 2% uranyl acetate.

Both peptides, P-GnRH and PT-GnRH, were synthesized using microwave-assisted Fmoc-SPPS on rink amide MBHA resin [31] with 4-pentynoyl acid coupled to their N-terminus. The synthesized alkyne-modified peptides were conjugated to azide-containing PMA using the copper(I)-catalyzed alkyne-azide cycloaddition (CuAAC) “click” reaction [25]. Conjugates **1** and **2** were both self-assembled via solvent exchange (DMF-water), then extensively dialyzed for three days against water to remove unreacted peptide and residual copper. The substitution efficacy of the conjugation was determined through element microanalysis by comparison of the C/N ratio of unsubstituted PMA with nitrogen reach conjugate (Supporting Information Table S1), as previously reported [37,38]. Nearly quantitative substitution was achieved for both **1** and **2**. Conjugate **1** was freeze-dried with trehalose (**1-Tr**) and/or incorporated with liposomes (**L-1** and **L-1-Tr**). DLS analysis showed that **1** and **2** formed relatively monodisperse nanoparticles (Table 1, Supporting Information Figure S3). Nanoparticle size was further confirmed by TEM (Figure 3). As expected, the addition of trehalose increased nanoparticle size.

### 3.2. Immunization Study in Mice

**Figure 4:**
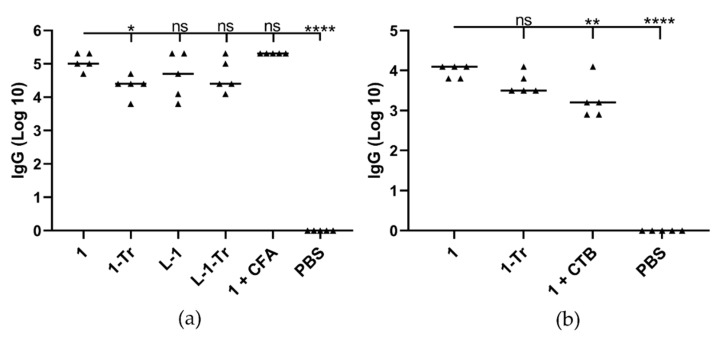
GnRH-specific antibody responses. (**a**) GnRH-specific serum IgG titers following single immunization by subcutaneous injection, and (**b**) GnRH-specific serum IgG titers following single oral immunization. Not significant (ns) *p* > 0.05, (*) *p* < 0.05, (**) *p* < 0.01, (****) *p* < 0.0001.

Six-week-old mice (C57/BL6 female mice, 5 per group) were subcutaneously immunized with a single dose of **1**, **1-Tr**, **L-1**, **L-1-Tr**, **1+CFA** (complete Freund’s adjuvant) as a positive control, and PBS as a negative control. All test and positive control mice received the same dose of **1** (30 µg in 30 µL PBS), regardless of formulation composition. Serum IgG antibody titers were measured on day 13 post-immunization (Figure 4a, Supporting Information Figure S4). Vaccine candidate **1** induced the same level of anti-GnRH antibody titers as strong commercial adjuvant (**1+CFA**) after single immunization. Conjugate **1** produced slightly higher antibody titers than liposome- and trehalose-containing formulations (**L-1** and **1-Tr**). The use of cryoprotectant (trehalose) reduced both conjugate and liposome efficacy (**1** vs. **1-Tr**, and **L-1** vs. **L-1-Tr**). As liposomal formulations (**L-1**, **L-1-Tr**) did not induce stronger immune responses than **1** following subcutaneous immunization, only conjugate **1** was used for oral immunization, with and without cryoprotectant (Figure 4b, Supporting Information Figure S5). Interestingly, mice immunized with **1** produced higher IgG titers than **1** adjuvanted with CTB after a single immunization, while the addition of cryoprotectant (**1-Tr**) did not significantly reduce antibody titers. It is important to note that formulations with cryoprotectant (**1-Tr** and **L-1-Tr**) were freeze-dried with trehalose, then resuspended; thus, they were not simple mixtures of **1** and **L-1** with trehalose.

### 3.3. Immunization Study in Pigs

**Figure 5:**
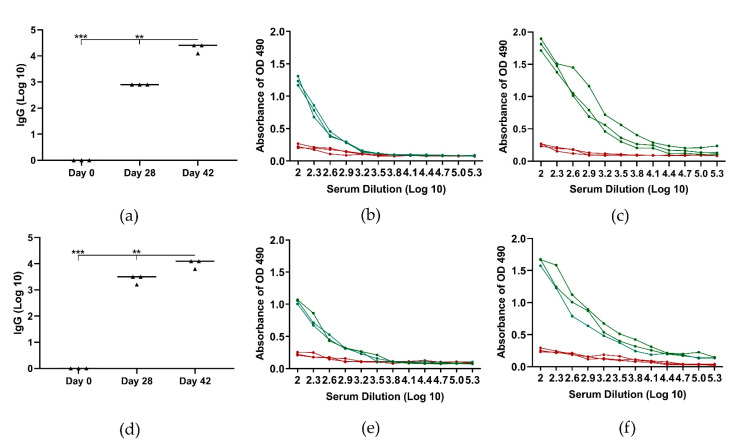
GnRH-specific antibody (IgG) response following intramuscular (**a**–**c**) and oral (**d**–**f**) administration of conjugate **2** in pigs: (**a**) GnRH-specific serum IgG titers before (day 0) and at day 28 and day 42; (**) *p* < 0.01, (***) *p* < 0.001. (**b**) optical density (OD) values from ELISA analysis of log serum dilutions at day 28; (**c**) OD values from ELISA analysis of log serum dilutions at day 42; (**d**) GnRH-specific serum IgG titers before (day 0) and two weeks after each oral immunization (day 28 and day 42); (**) *p* < 0.01, (***) *p* < 0.001. (**e**) OD values from ELISA analysis of log serum dilutions at day 28; (**f**) OD values from ELISA analysis of log serum dilutions at day 42. The green lines represent OD values of sera from immunized pigs, while the red lines are OD values of pig sera prior to immunization.

Six Female Large white pigs were randomly assigned to two groups; the first group received vaccine **2** (3 mg per pig) intramuscularly, the second group received **2** (30 mg per pig) by oral gavage (Figure 5) at day 0 and 28. Pigs intramuscularly immunized with conjugate **2** produced high antibody levels on day 28 (Figure 5a,b) and antibody production increased further after the second immunization (day 42, Figure 5a,c). Compound **2** also induced the production of GnRH-specific antibodies following oral immunization, with levels only slightly lower than those induced by intramuscular immunization (Figure 5d–f). Pig body weight increased during experimentation, from 92.6 ± 1.9 kg before immunization to 120 ± 2.0 kg at euthanasia (day 42) for the intramuscular group, and 94.3 ± 1.9 kg to 124 ± 2.2 kg for the oral group.

## 4. Discussion

GnRH is the most common target for contraceptive vaccines. As an endogenous peptide, GnRH does not normally induce an immune response in animals. However, once GnRH is conjugated to appropriate T helper epitope [39] and combined with a delivery system or adjuvant, it can generate hormone-specific antibodies [40,41]. In the present study, T helper epitope, PADRE (P), and pig T helper (PT) epitope were conjugated to GnRH, producing P-GnRH and PT-GnRH, respectively. Universal P T helper was chosen because it is well-known for its ability to generate T helper cell immune responses in humans and mice [42,43]. Similarly, PT from classical swine fever virus (CSFV) non-structural protein, NS3, has been proven to deliver T helper responses in pigs [44,45]. The crucial role of the T-helper has been confirmed during development of vaccines against classical swine fever virus [44,46,47,48,49,50], porcine reproductive and respiratory syndrome virus [49,51], African swine fever virus [52], and foot and mouth disease virus [49].

A variety of adjuvants and delivery systems have been used to enhance immune responses against peptide antigens [53,54]. However, many of these proved to be toxic, or poorly effective, especially for oral administration. Recently, polymer-based self-adjuvanting delivery systems have been extensively investigated [27,55,56], and polyacrylate delivery systems (including PMA) were especially efficient. They were able to protect peptide antigens against enzymatic degradation [57]; induce strong immune responses, both humoral [24,25,26] and cellular [34]; and they were effective after a single immunization [26,58] and through oral administration [30,59,60]. Immunogenicity of these polymeric systems is typically explained by their ability to form amphiphilic products upon conjugation to hydrophilic peptide antigen. The formed conjugates can then self-assemble into nanoparticles, which can mimic natural infection (due to size, charge, and similarities in antigen presentation) [61].

We examined PMA for its ability to form nanoparticles following conjugation to GnRH/T helper peptides. Both conjugates **1** and **2** (Figure 1 and Figure 2) self-assembled into nanoparticles, as expected. Once the nanoparticles were freeze-dried for long-term storage, they could not be re-solubilized under aqueous conditions. Therefore, trehalose was added to the formulation (**1-Tr**) as a cryoprotectant. Trehalose’s ability to protect biomolecules in aqueous solution from degradation during freeze-drying has been widely reported [62,63]. Several **1**: trehalose ratios were examined; ratio 1:5 produced the smallest nanoparticles with the lowest PDI upon freeze-drying and reconstitution in PBS/water. Interestingly, the PDI of **1-Tr** (0.19) decreased in comparison to **1** (0.36), suggesting that trehalose also stabilized nanoparticles in solution and reduced aggregation.

Liposomes were extensively used for peptide-based vaccine delivery systems [64]. Liposomes could protect peptide antigens from degradation and act as an adjuvant/immunostimulant [65]. Significantly enhanced anticancer immunogenicity was reported when liposomes were applied as a carrier for polyacrylate-peptide antigen conjugate [29]. The encapsulation of conjugate **1** with liposomes (**L-1**) was expected to improve vaccine immunogenicity. The liposomes were also formulated with trehalose (**L-1-Tr**) aiming at the long-term storage of the vaccine.

Initially, nanoparticles **1**, **1-Tr**, **L-1**, and **L-1-Tr** were examined in mice following single tail-base subcutaneous injection. Self-adjuvanting nanoparticles **1** (62 nm) were more effective than the liposomal formulation **L-1** (145 nm) most likely due to their smaller particle size (Table 1, Figure 4a). The addition of trehalose slightly reduced antibody titers; this was most likely also associated with the resulting increase in particle size [66,67]. Importantly, nanoparticles **1** were as effective as *Mycobacterium*-derived adjuvant (CFA) in inducing anti-GnRH IgG titers.

The most potent system developed in this study, **1**, and its trehalose formulation, **1+Tr**, were used to examine the vaccine’s capacity to induce antibody production following oral immunization. CTB is a standard commercial adjuvant for oral vaccine delivery [68]. Interestingly, when CTB was mixed with **1**, lower systemic IgG titers were produced in comparison to those induced by **1** alone (Figure 4b). This phenomenon was previously observed for polymeric conjugates [30] and might be related to CTB’s ability to provoke tolerance upon oral administration [68,69,70]. Again, the addition of trehalose only slightly reduced IgG titers, demonstrating that trehalose can be applied to provide long-term storage of the formulation in freeze-dried form.

Conjugate **1**, upon T helper replacement, was also able to self-assemble into nanoparticles **2** (141 nm) and generate strong immune responses after single subcutaneous and oral immunization of pigs (Figure 5). The level of anti-GnRH IgG titers induced by **1** and **2** were higher than those induced by the lipopeptide GnRH vaccine candidate upon intramuscular immunization of mice and rams [40,41]. Notably, these anti-GnRH IgG titers were high enough to greatly reduce testicle dimeter in rams [40]. The viability of oral immunization has been confirmed during development of swine vaccines against infectious diseases. Oral vaccines distributed as bait have produced antibody responses against classical swine fever virus [44,71,72,73,74,75,76], African swine fever virus [77,78] and rabies virus [79].

It is important to mention that commercial anti-GnRH vaccines (Improvac™ and GonaCon™) are available [80,81]. However, Improvac™ requires several doses to stimulate high antibody titers, and both are effective only upon intramuscular delivery [80,82]. Thus, we demonstrated for the first time that a GnRH vaccine can induce strong immune responses without adjuvant and following oral administration. Development of an effective orally administered vaccine is especially challenging, as (a) peptides are not normally recognized as antigens in the gastrointestinal tract; (b) they are rapidly degraded by proteases; (c) tight epithelial cellular junctions and thick mucus prevent systemic absorption of peptides; and (d) tolerance to peptide antigens is easily developed [83].

Antibody titers following vaccination with the developed formulations were very consistent between pigs immunized with the same agent, showing the ability of PMA and T helper to induce immunity in outbred populations. Importantly, vaccine **2** was well-tolerated by the pigs and no site reactions were observed. The body weight of vaccinated pigs increased as expected for healthy animals. Further studies are needed to optimize vaccine **2** immunogenicity and examine its antifertility efficacy. The next phase of testing may also involve the incorporation of the helper T-cell-based antifertility vaccine (**2**) in baits that have been proven to be effective in wild boar with disease vaccines [84].

## 5. Conclusions

Currently, there are no GnRH-based oral vaccines or highly effective and safe oral adjuvants approved for animal or human use. In this study, we demonstrated that polymer-based delivery systems can induce the production of high levels of systemic anti-GnRH IgG after a single immunization in mice and pigs. Moreover, efficient antibody production was achieved via oral immunization and no adverse effects were observed in immunized animals.

We produced the first adjuvant-free, orally active vaccine candidate against GnRH. The vaccine was developed for fertility control of pigs/boars; however, it may also be adaptable to provide a treatment for hormone-dependent cancers in humans (e.g., prostate, breast, endometrial, and ovarian cancers).

## Figures and Tables

**Table 1:** Characterization of the vaccine candidates.

Compound	Particle Size (nm)	Polydispersity Index (PDI)
**1**	62 ± 1	0.36 ± 0.03
**1-Tr**	140 ± 4	0.19 ± 0.07
**L-0**	99 ± 1	0.14 ± 0.03
**L-1**	146 ± 2	0.19 ± 0.04
**L-1-Tr**	171 ± 2	0.22 ± 0.09
**2**	141 ± 5	0.08 ± 0.02

## Data Availability

The data presented in this study are available on request from the corresponding author.

## Supplementary Materials

The following are available online at https://www.mdpi.com/article/10.3390/pharmaceutics13071081/s1, Figure S1: ESI-MS spectrum of the 4-petynoyl derivative of GnRH-PADRE peptide and analytical image, Figure S2: ESI-MS spectrum of the 4-petynoyl derivative of GnRH-PT peptide and analytical image, Figure S3: Particle size distribution by intensity and polydispersity index (PDI), Figure S4: GnRH-Specific antibody (IgG) responses following subcutanious administration in mouse, Figure S5: GnRH-specific antibody (IgG) responses following oral administration in mouse, Figure S6: Female Large White pigs were used for the injection and oral vaccine study (left panel). Oral vaccine was delivered by gavage using a Shoof Luer-Lock (206 285) drench nozzle (right panel), Table S1: Element microanalysis for PMA, PMA-P-GnRH, and PMA-PT-GnRH.
