# Supplementary material for: Polyacrylate-GnRH Peptide Conjugate as an Oral Contraceptive Vaccine Candidate
Source: Pharmaceutics. 2021 Jul 15;13(7):1081. doi: 10.3390/pharmaceutics13071081 (PMC8308917; doi:10.3390/pharmaceutics13071081)
Supplement: Supplementary file 1 [file pharmaceutics-13-01081-s001.zip › pharmaceutics-1273720 - supplementary.pdf]

Supplementary Materials: Polyacrylate-GnRH Peptide Conjugate as an Oral Contraceptive Vaccine Candidate

Mohammad Omer Faruck, Prashamsa Koirala, Jieru Yang, Michael J. D'Occhio, Mariusz Skwarczynski and Istvan Toth

Table S1. Element microanalysis of PMA, **1** and **2**.

Conjugates	Theorital Value			Experimental Value		
	N%	C%	N/C ratio	N%	C%	N/C ratio
PMA	1.53	55.00	0.028	1.51	54.80	0.028
PMA-P-GnRH (**1**)	10.27	49.81	0.206	10.19	49.80	0.204
PMA-PT-GnRH (**2**)	10.84	55.18	0.197	10.73	54.89	0.196

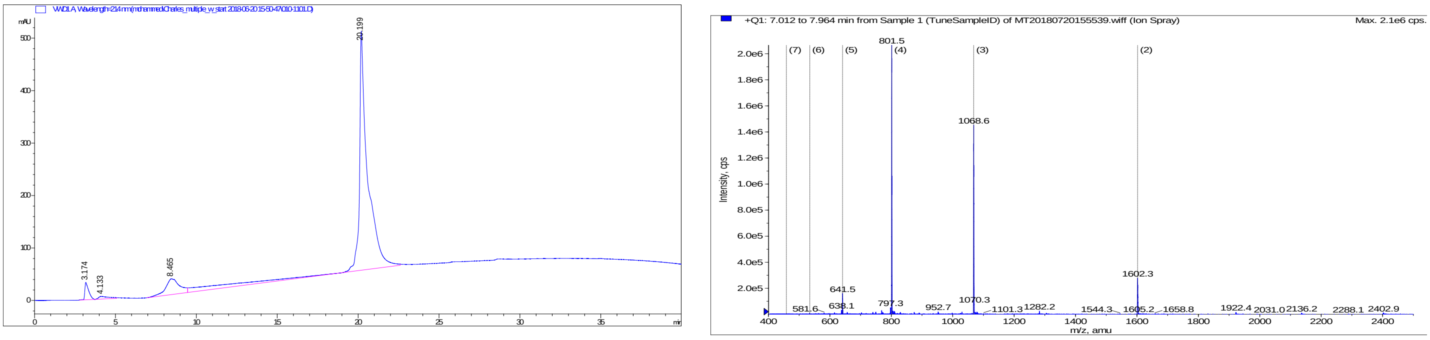

(a) (b)

Figure S1. Analysis of purified compound PADRE-GnRH with alkyne moiety (**a**) HPLC (**b**)ESI-MS. HPLC, C18 column, t_R 20.1 min, purity 96%, yield 43%. Analytical RP-HPLC graphs show pure compounds in single peak. The mass from these peaks, matched to the desired.

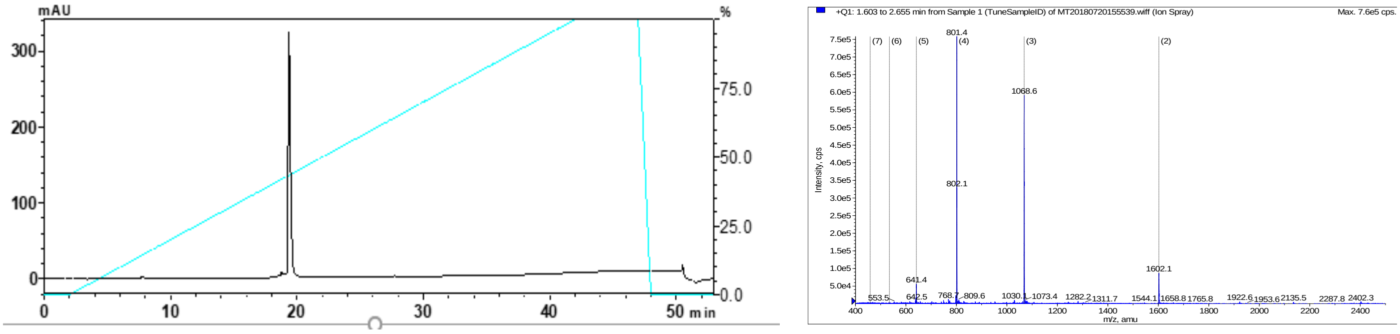

(a) (b)

Figure S2. Analysis of purified compound pig T helper-GnRH with alkyne moiety (**a**) HPLC (**b**)ESI-MS. HPLC, C18 column, HPLC C18 column, t 19.8 min, purity 99%, yield 43% Analytical RP-HPLC graphs show pure compounds in single peak. The mass from these peaks, matched to the desired compounds in ESI-MS.

Particle size by Intensity **Particle Size by Number**

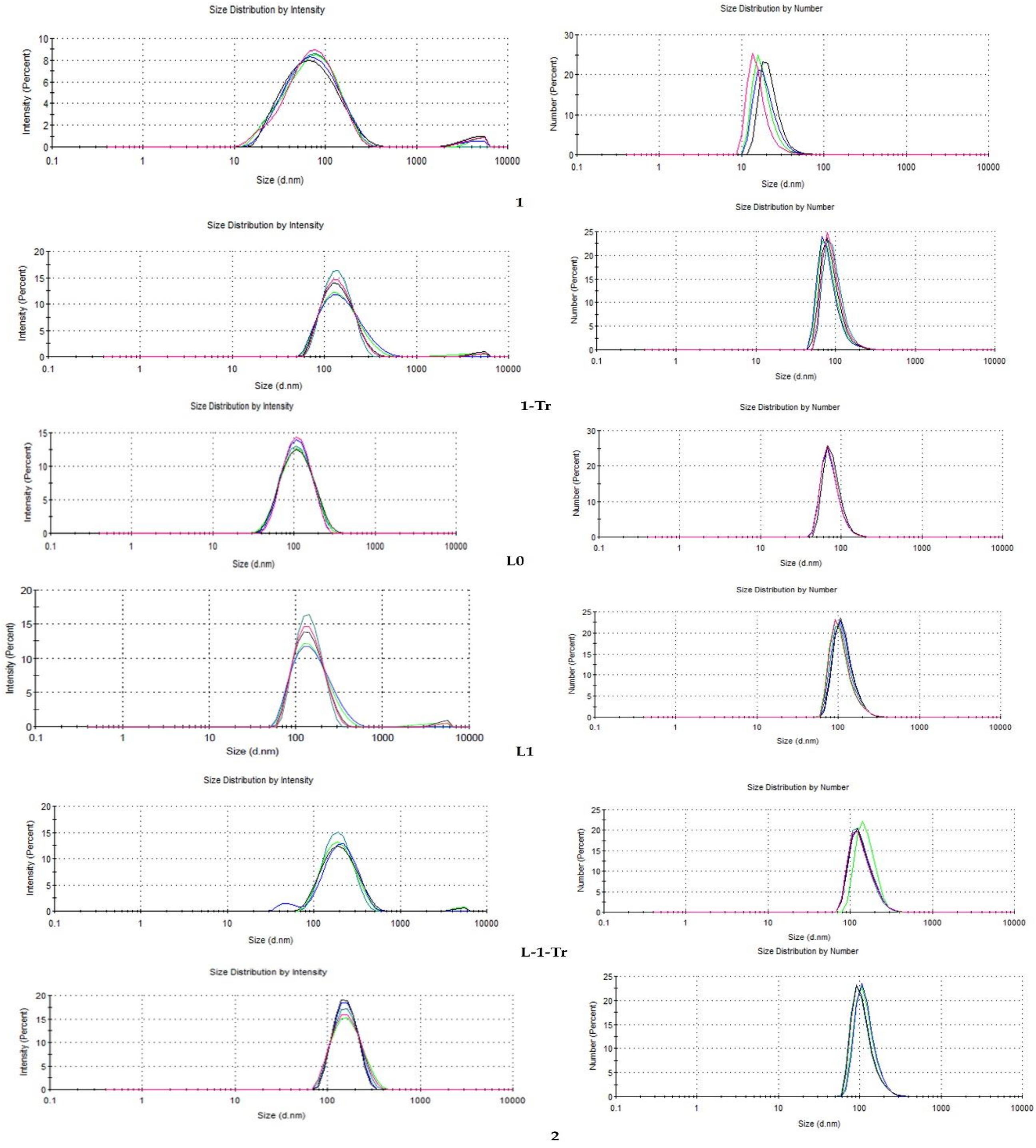

Figure S3. Particle size distribution by intensity and Particle size distribution by number of all vaccine compounds. **1** particle size 62 ±1 nm PDI 0.36 ± 0.03, compound **1-Tr** particle size 140 ± 4 nm, PDI 0.19 ± 0.07, **L-0** particle size 99 ± 1 nm, PDI 0.14 ± 0.03, **L-1** particle size 146 ± 2, PDI 0.19 ± 0.04, **L-1-Tr** particle size 171 ± 2 nm, PDI 0.22 ± 0.09, Compound **2** particle size 141 ± 5 nm, PDI 0.08 ± 0.02.

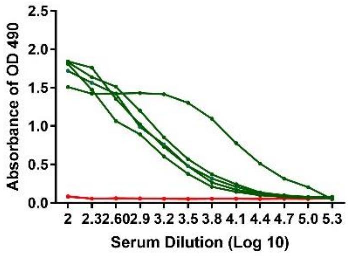

(a)

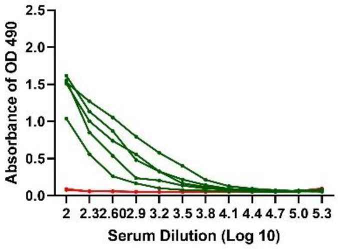

(b)

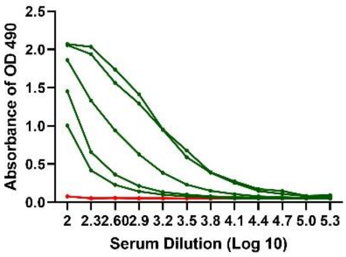

(c)

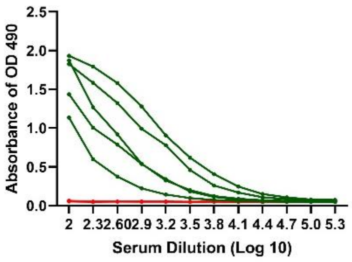

(d)

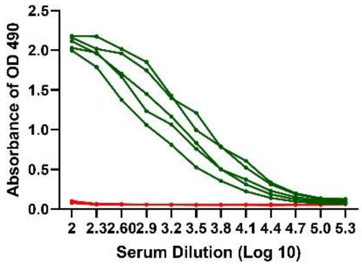

(e)

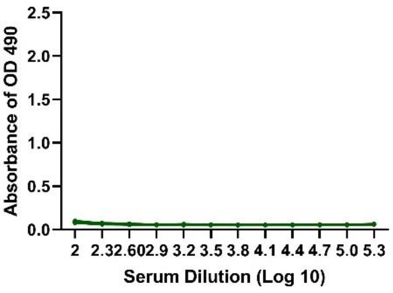

(f)

Figure S4. GnRH-Specific antibody (IgG) responses following subcutaneous administration of the vaccine candidates in mice. Optical density (OD) value from ELISA analysis of log serum dilution after single immunization (**a**) 1, (**b**) 1-Tr, (**c**) L-1, (**d**) L-1-Tr, (**e**) 1+CFA, (**f**) PBS.

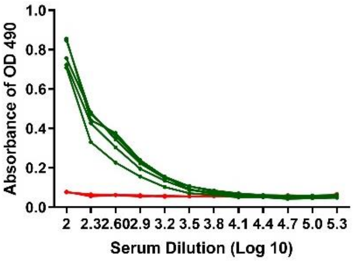

(a)

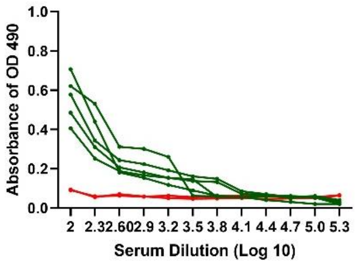

(b)

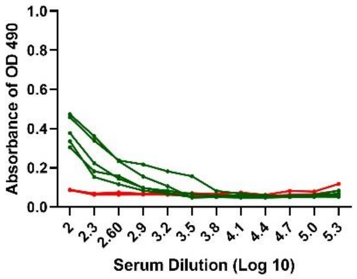

(c)

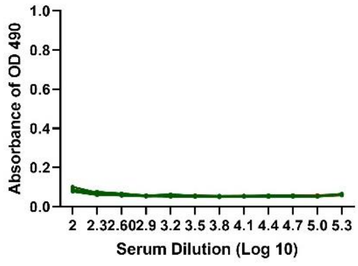

(d)

Figure S5. GnRH-Specific antibody (IgG) responses following subcutaneous administration of the vaccine candidates in mice. Optical density (OD) value from ELISA analysis of log serum dilution after single immunization (**a**) **1**, (**b**) **1-Tr**, (**c**) **1**+CTB, (**d**) PBS.

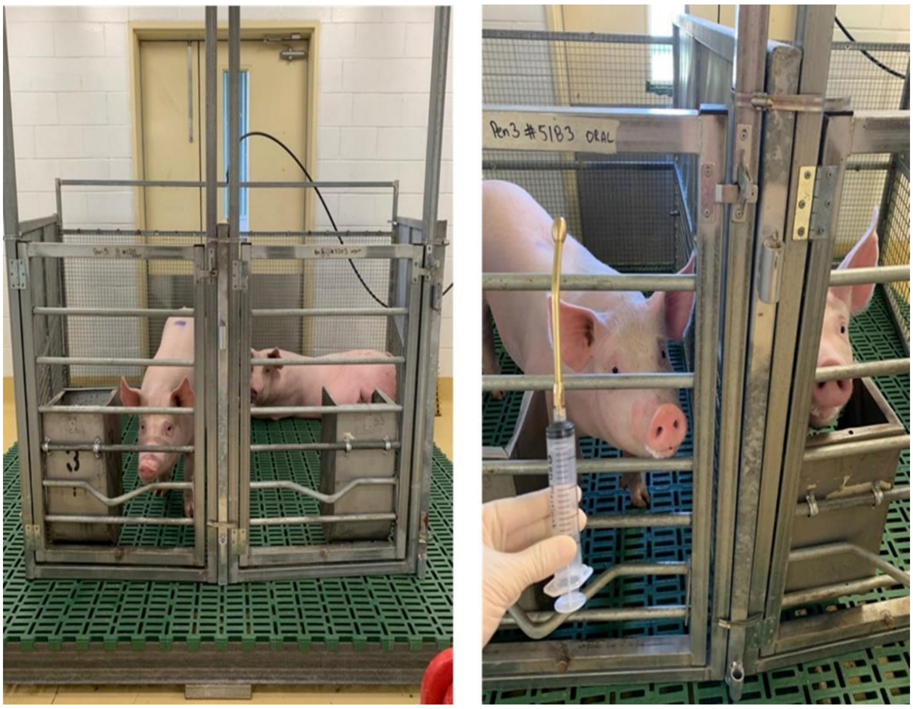

Figure S6. Female Large White pigs were used for the injection and oral vaccine study (left panel). Oral vaccine was delivered by gavage using a Shoof Luer-Lock (206 285) drench nozzle (right panel).
